# Reprogramming mitochondrial metabolism of macrophages by miRNA-released microporous coatings to prevent peri-implantitis

**DOI:** 10.1186/s12951-023-02244-z

**Published:** 2023-12-17

**Authors:** Hongming Zhang, Yun Yuan, Hanxiao Xue, Runping Yu, Xiayue Jin, Xiaolin Wu, Hui Huang

**Affiliations:** 1 Department of Prosthodontics, Ninth People’s Hospital Affiliated to Shanghai Jiao Tong University School of Medicine, No. 500 Quxi Rd, Huangpu District, Shanghai, China; 2 College of Stomatology, Shanghai Jiao Tong University; National Center for Stomatology; National Clinical Research Center for Oral Diseases, Shanghai, China; 3 Shanghai Key Laboratory of Stomatology, Shanghai Research Institute of Stomatology, Shanghai, China

**Keywords:** Peri-implantitis, Macrophages, Dental implants, Mitochondrial metabolism

## Abstract

Although various new biomaterials have enriched the methods for peri-implant inflammation treatment, their efficacy is still debated, and secondary operations on the implant area have also caused pain for patients. Recently, strategies that regulate macrophage polarization to prevent or even treat peri-implantitis have attracted increasing attention. Here, we prepared a laser-drilled and covered with metal organic framework-miR-27a agomir nanomembrane (L-MOF-agomir) implant, which could load and sustain the release of miR-27a agomir. In vitro, the L-MOF-agomir titanium plate promoted the repolarization of LPS-stimulated macrophages from M1 to M2, and the macrophage culture supernatant promoted BMSCs osteogenesis. In a ligation-induced rat peri-implantitis model, the L-MOF-agomir implants featured strong immunomodulatory activity of macrophage polarization and alleviated ligation-induced bone resorption. The mechanism of repolarization function may be that the L-MOF-agomir implants promote the macrophage mitochondrial function and metabolism reprogramming from glycolysis to oxidative phosphorylation. Our study demonstrates the feasibility of targeting cell metabolism to regulate macrophage immunity for peri-implantitis inhibition and provides a new perspective for the development of novel multifunctional implants.

**Graphical Abstract:**

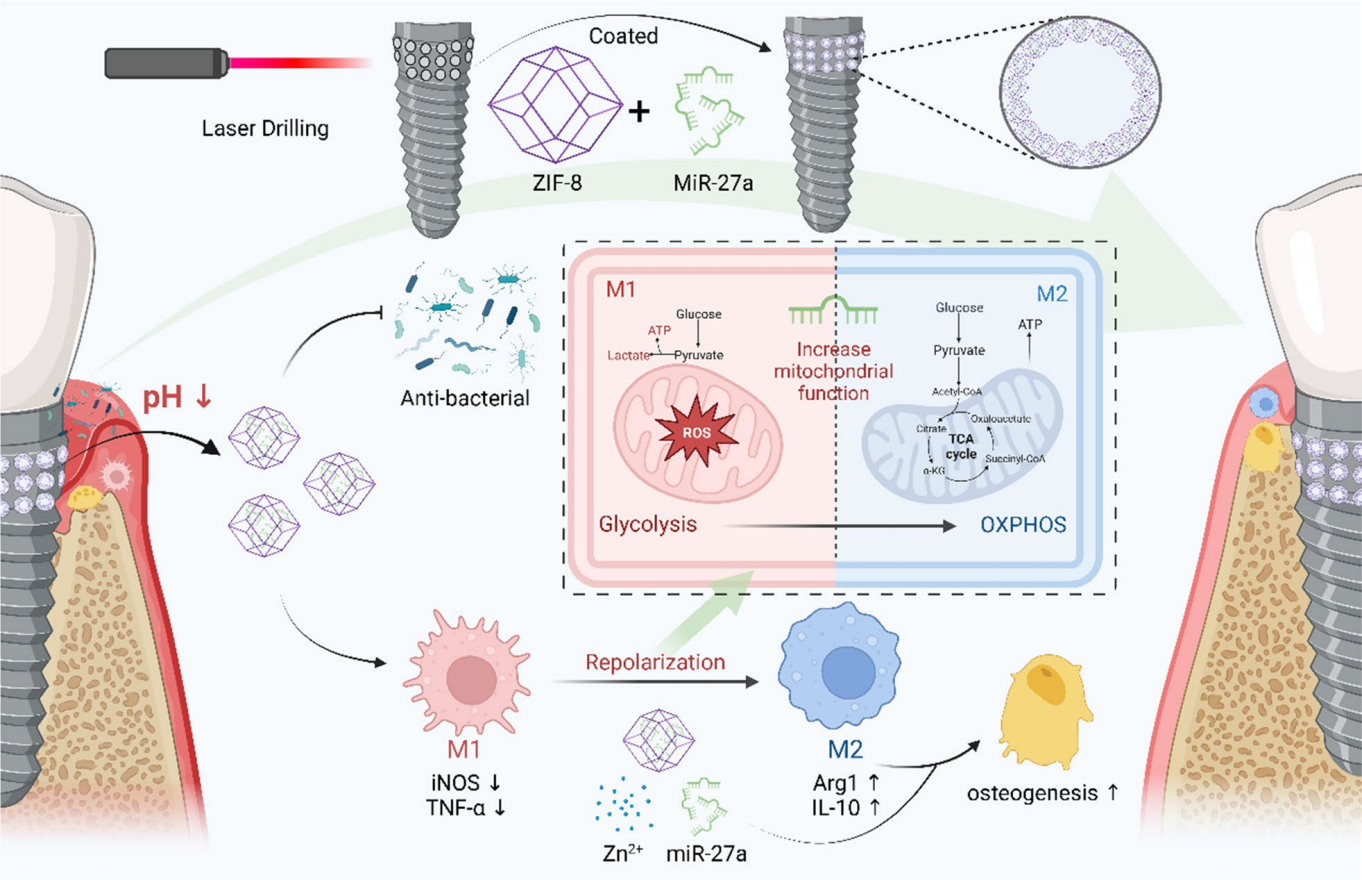

**Supplementary Information:**

The online version contains supplementary material available at 10.1186/s12951-023-02244-z.

## Introduction

Peri-implantitis is a biological complication that occurs in the peri-implant tissue, characterized by inflammation of the connective tissue around the implant and progressive resorption of the supporting alveolar bone [1]. Unlike natural periodontal tissue, the implant-soft tissue interface has a weaker defense against bacterial invasion [2]. To promote early formation of the osseointegration interface, inhibiting inflammation and bone resorption in peri-implantitis is emerging as a research concern. Studies on the treatment of peri-implantitis have revealed that surgical treatment seems to improve the treatment effect of peri-implantitis, but usually requires flap surgery and resection therapy, which is painful and costly [3]. However, nonsurgical treatment has limited therapeutic effects, which may be due to limited access to the implant surface, unsustainable duration of action, persistent stimulants, inability to restore the balance of the immune microenvironment, and difficulty in transforming osteoclastic bone resorption into osteogenic processes [4, 5]. Considering that excessive inflammation is more directly related to bone resorption, bone therapy strategies that target local immune microenvironment regulation may be an effective approach, but implant-based immune regulation strategies have not been seen.

As an innate immune cell, macrophage can polarize toward different functional phenotypes M1/M2 in response to a variety of biophysical/biochemical signals [6, 7]. Pathologic studies have found that there is a larger lesion area, a larger number and proportion of macrophages and vascular infiltration around the implant in peri-implantitis [8]. The M1/M2 ratio in granulation tissue of peri-implantitis was significantly correlated with the probing depth, and the imbalance of M1/M2 may play a key role in inflammation [9]. Editing macrophage polarization is emerging as a new therapeutic approach [10, 11]. In many inflammatory diseases, the treatment effect benefits from the polarization to M2 macrophages. Although many biomaterial strategies effectively modulate the direction of macrophage polarization, the repolarization of M1 to M2 appears to be the current challenge. M1 macrophages fail to convert into M2 cells upon IL-4 exposure in vitro and in vivo. In contrast, M2 macrophages are more plastic and readily repolarized into M1 state. Hence, effective strategies are needed to regulate macrophage polarization and repolarization.

MicroRNAs (miRNAs), as potent molecular managers, may simultaneously regulate multiple endogenous processes, such as inflammation and bone remodeling. Our previous study found that miR-27a is highly conserved and downregulated in peri-implantitis [12]. Upregulated expression of miR-27a positively regulated osteogenesis-angiogenesis coupling by ameliorating TNF-α inhibition of bone formation in vitro. Furthermore, we constructed hydroxyapatite scaffolds loaded with miR-27a overexpressed mesenchymal stem cells to repair the bone defect around the implant [13]. Although certain effects have been achieved, the overexpression of miR-27a with viruses has brought some concerns about biosafety, and the implantation of mesenchymal stem cells has also brought concerns about immune rejection. The formulation of miRNAs into nanoscale delivery vehicles represents an effective strategy for enhancing bioavailability and biocompatibility [14, 15]. This general approach has helped to greatly improve therapeutic outcomes for miRNA therapies [16, 17]. In addition, studies have found that after LPS stimulus, the expression of miR-27a was downregulated in macrophage [18], and upregulation of miR-27a alleviated LPS-induced acute lung injury [19]. In breast cancer, miR-27a promotes immune escape by regulating PD-L1 expression in macrophages [20]. Therefore, miR-27a may play an important role in M2 polarization, and regulating the expression of miR-27a to regulate the M1/M2 balance in peri-implantitis may be a feasible strategy.

The need for accelerating osseointegration for rapid loading and for decreasing the occurrence of peri-implantitis has encouraged advancements in implant surface modification. Physical modification can directly affect both osseointegration and biofilm formation by modifying the surface roughness. The micromechanical changes lead to improved secondary integration, including bone growth, turnover, remodeling, and overall interlocking of bone at the implant surface, whose diameter often ranges from 1 to 100 μm. Chemical modifications, such as discrete crystalline deposition, anodic oxidation, and photofunctionalization, provide a hydrophilic surface and release metal ions to affect osteogenesis and bacterial adhesion. More direct stimulation of osseointegration and mitigation of biofilm formation can be accomplished by specific biological coatings, which can regulate cell proliferation, differentiation and functions by loading cytokines, proteins and genes. Considering the complexity of the peri-implant cell-bacterial balance and the difference between the osteogenic environment and the inflammatory environment around the implant, a composite modification with different functions in different environments is needed.

In this work, we developed a micro-nano implant with responsive and sustained release of miR-27a to modulate macrophage polarization by targeting immunometabolism and mitochondrial function for the prevention and treatment of peri-implantitis. Specifically, a small osteogenic micropore with 100 μm in diameter [21, 22] was drilled by femtosecond laser, which has a mature application in the surface modification of implants and can modify the morphology without changing the crystal phase [23]. Then, a miRNA-loaded MOF membrane was coated on the wall of the micropore. The zeolitic imidazolate framework-8 (ZIF-8) MOFs used in this work have minimal toxicity and have previously been used for the delivery of biomolecules [24, 25]. The structural integrity of the MOF scaffolds is pH dependent [26, 27], a property we leveraged to achieve responsive release of miR-27a in inflammatory environments. The immunomodulatory and osteogenic functions of laser-MOF-miR-27a agomir (L-MOF-agomir) implant was tested in a rat peri-implantitis model, and the macrophage repolarization mechanism was investigated using L-MOF-agomir titanium plates in vitro cell culture systems. This study offers valuable information for exploring multifunctional implants, and provides a novel perspective for the treatment of peri-implantitis.

## Materials and methods

### Preparation of nanoparticles and coated titanium

To prepare the MOF-agomir nanoparticles, an appropriate amount of agomir-27a (Ribobio) was premixed in a 60 mM 2-methylimidazole (Macklin, Shanghai, China) solution to achieve different concentrations of agomir input. Then solution of 1 mM zinc acetate dihydrate (Macklin) and premixed solution was mixed with equal volume. The final mixture was vortexed for 10 s and left undisturbed at 30 °C for 6 h. Agomir-cyanine 3 (Cy3)-labeled negative control (Ribobio, Guangzhou, China) was used for characterization studies. MOF-agomir nanoparticles were isolated by centrifugation at 10,000 rpm for 10 min and rinsed with double distilled water (ddH_2_O) and methanol three times.

For L-MOF-agomir titanium preparation, commercially available TC4 titanium plates (diameter: 30 mm) were ultrasonically cleaned with acetone, ethanol and ddH_2_O three times, respectively. Then the plates were drilled using a femtosecond laser (INNO Machining Co., Ltd., Changzhou, China), followed by ultrasonic cleaning with acetone, ethanol and ddH_2_O to remove impurities. The secondary growth method was performed to prepare MOF-agomir film on titanium. Typically, the MOF-agomir nanoparticles obtained above were redispersed in fresh methanol to form a seed suspension (0.1 wt%). The laser drilled titanium plates (L) were dipped into the seed suspension for 10 s and allowed to dry at RT for 5 min. This procedure was repeated once to improve coverage. The samples were placed horizontally in appropriate orifice plates with freshly prepared secondary growth solution, which was produced through mixing and vortexing 60 mM 2-methylimidazole, 100 nM agomir-27a and 1 mM zinc acetate dihydrate solutions for 10 s. Then, the orifice plates were left undisturbed at 30 °C for 6 h. After that, the titanium plates were rinsed with ddH_2_O and methanol three times and immersed in phosphate buffered saline (PBS).

### Nanoparticle and titanium characterization

The morphology of the nanoparticles and energy disperse spectroscopy (EDS) images were observed and photographed on a JEOL JEM 2100F transmission electron microscope (TEM). To confirm the loading of the agomir in the MOF particles, we prepared MOF-agomir-Cy3 particles and observed the colocation images using a fluorescence microscope (Axio Scope A1, Zeiss, German). To quantify the encapsulation rate and release of agomir from MOF-agomir particles and L-MOF-agomir titanium plates, agomir-Cy3 was loaded in MOF and then the samples were resuspended in PBS at pH 5.5, pH 6.5 or pH 7.4. At different predetermined time points, samples from different groups were centrifuged to pellet the nanoparticles, and the fluorescence of the Cy3 dye (excitation/emission = 550/570 nm) in the supernatant was measured using Tecan Spark 10 M multimode microplate reader. According to our results, 100 nM agomir-27a was used for subsequent studies. Nanoparticle size was measured with LA-950 laser scattering particle size distribution analyzer after ultrasonic dispersion, and zeta potential was measured with Malvern Zetasizer Nano ZS90. The surface morphology and chemical analysis of the titanium samples were observed and measured with scanning electron microscope (SEM) and EDS integrated machine (Phenom Pro X, Phenom Scientific). EDS was performed using line scanning on the wall of the micropore at least 6 times.

### Cell culture and cytocompatibility evaluation

Bone marrow stem cells (BMSCs) were isolated from the bone marrow of Sprague–Dawley rats as reported previously. Briefly, bone marrow was flushed from the tibias and femurs with medium. The cells were centrifuged, resuspended, and cultured in Dulbecco’s Modified Eagle Medium (DMEM; HyClone) containing 10% fetal bovine serum (FBS, Gibco) and 1% penicillin and streptomycin (Gibco) at 37 °C in a 5% CO_2_ atmosphere. The medium was changed every 3 days and the subculture was carried out when the confluence reached 80%. Third passage BMSCs were used for all cell experiments. RAW 264.7 cells were obtained from American Type Culture Collection (ATCC). RAW 264.7 cells were cultured in DMEM supplemented with 10% FBS and 1% penicillin and streptomycin at 37 °C under 5% CO_2_.

The viability of cells was analyzed using Calcein/PI Live/Dead Viability/Cytotoxicity Assay Kit (Beyotime, Shanghai, China) and Cell Counting Kit-8 (CCK8, Dojindo, Kyushu, Japan) according to the manufacturer’s instructions. Briefly, after a culture period of appropriate time on the titanium plates, each sample was incubated with calcein-AM and propidium iodide for 30 min at 37 °C in the dark. Then each sample was rinsed and examined with fluorescence microscope (Axio Scope A1, Zeiss, German). For the CCK8 test, after the appropriate culture time, 10% of the medium volume of reagent was added and incubated for 1 h at 37 °C. Subsequently, the supernatant was collected for examination with a microplate reader (Tecan Spark 10 M, Switzerland) at 450 nm. The cell morphology and arrangement on the surface of titanium samples were observed with SEM (Phenom Pro X, Phenom Scientific). The relative miR-27a expression of BMSCs and RAW 264.7 cells on samples was quantified using real-time quantitative polymerase chain reaction (RT-qPCR). At day 1, 3 and 7, total RNA was isolated from the cells prepared above using TRIzol reagent (Invitrogen, Carlsbad, CA, USA) according to the manufacturer’s instructions, and the RNA concentration and purity were determined using a NanoDrop spectrophotometer (Allsheng, Hangzhou, China). Total RNA (1000 ng) was reverse transcribed into complementary DNA (cDNA) using Hifair® II 1st Strand cDNA Synthesis SuperMix (Yeasen Biotechnology Co., Ltd., Shanghai, China). Then, quantitative real-time polymerase chain reaction (qRT‒PCR) was performed using a Roche LightCycler 480 II. The initial denaturation process was 10 min at 95 °C, followed by 40 cycles of 95 °C for 10 s and 60 °C for 60 s.

### In vitro macrophage repolarization and BMSC differentiation on titanium samples

RAW 264.7 cells were incubated with 100 ng ml^−1^ Porphyromonas gingivalis (P.g) lipopolysaccharide (LPS) for 24 h. Then the cells were incubated on the surface of L, L-MOF, and L-MOF-agomir titanium samples in pH 6.5 medium for 72 h to investigate the effects of the samples on macrophage repolarization. Then, the macrophages were detached, centrifuged, and cultured with complete medium for 72 h to collect the supernatant for further studies.

The supernatant was discarded, and the samples were rinsed with PBS 3 times. The cells were fixed with 4% paraformaldehyde for 20 min. PBS with 0.5% Triton X-100 was used for cell permeabilization treatment for 20 min. The cells were blocked with normal donkey serum (1:20) at 25 °C for 1 h and then incubated with antibodies against CD206 (rabbit polyclonal to mannose receptor, ab64693, 1:1000, Abcam) or iNOS (rabbit monoclonal to iNOS, ab178945, 1:1000, Abcam) at 4 °C overnight. After rinsing with PBS, the CD206 and iNOS antibodies were detected with Alexa Fluor 647-conjugated donkey anti-rabbit IgG (34213ES60, 1:200, Yeasen) and Alexa Fluor 488-conjugated donkey anti-rabbit IgG secondary antibodies (34206ES60, 1:200, Yeasen), respectively, at 25 °C for 1 h. The nuclei were stained with 4′,6-diamidino-2-phenylindole (DAPI, 40728ES03, 1:1000, Yeasen) at 25 °C for 5 min. Finally, a fluorescence microscope (Scope A1, Zeiss) was used for observation. The number of positive cells was calculated in 3 randomly selected views per sample, and six randomly selected samples were used for the statistical analysis.

The cells on the titanium samples were collected, centrifuged, and resuspended for flow cytometry. The single cells were incubated with 1 μl of Fixable Viability Stain 780 (BD Horizon, USA) in 1 ml of DPBS. After incubation with 2 μl of Fc-block, 5 μl of PE-cy7-conjugated anti-CD86 antibody was used to stain extracellular markers. After fixation and permeabilization the cells were incubated with 5 μl Alexa Fluor 647-conjugated anti-CD206 antibody. Finally, the cells were resuspended in 300 μl of flow cytometry staining buffer and subjected to flow cytometry on a flow cytometer (BD LSRFortessa X-20 cell analyzer, BD Biosciences, USA). Data were analyzed using FlowJo_V10 software (Ashland, OR, USA).

BMSCs were incubated on the surface of titanium samples directly with pH 6.5 medium or incubated with the supernatant of macrophages treated as described above. Anti-alkaline phosphatase antibody (rabbit monoclonal to ALP, ab224335, 1:1000, Abcam), Alexa Fluor 647-conjugated donkey anti-rabbit IgG (34213ES60, 1:200, Yeasen), iF 488 phalloidin (40736ES75, 1:1000, Yeasen) and DAPI were used for alkaline phosphatase, cytoskeleton, and nuclei of BMSCs cultured directly, respectively. Anti-RUNX2 antibody (rabbit monoclonal to RUNX2, 12556, 1:1000, Cell Signaling Technology), Alexa Fluor 488-conjugated donkey anti-rabbit IgG secondary antibodies (34206ES60, 1:200, Yeasen), iFluor 647 phalloidin (40762ES75, 1:1000, Yeasen) and DAPI (40728ES03, 1:1000, Yeasen) were used for marking BMSCs cultured with supernatant. Images were observed with a fluorescence microscope (Scope A1, Zeiss).

BMSCs were incubated with macrophage supernatant for 7 days and 28 days for ALP (BCIP/NBT Alkaline Phosphatase Color Development Kit, C3206, Yeasen) and alizarin red (Alizarin Red S Staining Kit for Osteogenesis, C0148S, Yeasen) staining, respectively.

QRT-PCR was used to analyze the polarization states of macrophages and the differentiation states of BMSCs according to the methods mentioned above. The primer sequences are listed in Additional file 2: Table S1.

### Evaluation of antibacterial properties in vitro

P.g (ATCC 33277) was incubated in brain heart infusion (BHI) broth with 5% goat blood at 37 °C for 2 days. Next, bacteria were resuspended in BHI and incubated on titanium plates at 1 × 10^6^ CFU ml^−1^ for 24 h. Bacterial growth was determined by measuring absorbance at 600 nm using a spectrophotometer (Agilent Cary 60, Agilent Technology, USA). Bacterial survival was determined with fluorescence staining with LIVE/DEAD BacLight bacterial viability kit (L13152, Invitrogen). Briefly, component A and component B were mixed in ddH_2_O and placed into samples at room temperature in the dark for 15 min. Finally, a fluorescence microscope (Scope A1, Zeiss) was used for observation. The bacterial morphology was observed with SEM (Phenom Pro X, Phenom Scientific).

### Mitochondrial morphology and functional evaluation of macrophage in vitro

RAW 264.7 cells were incubated with 100 ng ml^−1^ LPS for 24 h and then incubated on the titanium samples for 72 h. TEM (JEOL JEM 2100F, Japan) was used to investigate mitochondrial nanostructure and quantity. Reactive oxygen species (ROS) were investigated with ROS Assay Kit (S0033S, Beyotime, China). Briefly, macrophage was treated as described above and incubated with DCFH-DA (1:1000) at 37 °C for 20 min. Then the cells were rinsed with serum-free medium 3 times and observed with a fluorescence microscope (Scope A1, Zeiss). The Enhanced Mitochondrial Membrane Potential Assay Kit with JC-1 (1:200, C2003S, Beyotime, China) was used for mitochondrial state evaluation according to the manufacturer’s instructions. Here, the cells were rinsed with PBS 3 times and incubated with JC-1 staining working solution at 37 °C for 20 min. Then the cells were rinsed with JC-1 dilution buffer for 3 times and observed with a fluorescence microscope (Scope A1, Zeiss). The ATP content in macrophage was determined with an ATP Assay Kit (S0026, Beyotime, China) according to the manufacturer’s instructions. First, the cells were rinsed with PBS twice and lysed at 4 °C for 10 min. Then the cell lysates were collected and centrifuged at 12,000*g* for 5 min. The supernatant was collected and added to black 96-well plates containing ATP measure working solution and black 96-well plates containing BCA measure working solution. Finally, the ATP concentration and protein concentration were measured with a microplate reader (Tecan Spark 10 M, Switzerland) at luminescence and 562 nm, respectively. The ATP concentration was normalized to the protein concentration.

### Mitochondrial metabolism evaluation

RAW 264.7 cells were incubated with 100 ng ml^−1^ LPS for 24 h and then incubated on the titanium samples for 72 h. Then the cells were collected to investigate the metabolism states by measuring mitochondrial oxygen consumption rates (OCRs) and extracellular acidification rates (ECARs) with XF^e^96 Seahorse (Seahorse Bioscience, Agilent Technologies, USA). For the OCR experiment, the basal OCRs were determined first, and then 1 μM oligomycin (Oligo), 1 μM carbonyl cyanide-4 phenylhydrazone (FCCP) and 1 μM rotenone and antimycin A (ROT/AA) were sequentially added during the OCR measurements. The respiratory parameters were calculated as follows:

$$\begin{aligned} & {\text{Basal respiration}}\, = \,{\text{value measured before Oligo treatment}}; \\ & {\text{ATP production}}\, = \,{\text{value before Oligo treatment}}-{\text{value after Oligo treatment;}} \\ & {\text{Respiration capacity}}\, = \,{\text{value after FCCP treatment}}-{\text{value after ROT and AA treatment}}. \\ \end{aligned}$$

For ECARs experiments, the basal rate was determined and then 10 mM glucose, 1 μM Oligo and 1 μM 2-deoxy-glucose (2-DG) were sequentially added. The glycolysis parameters were calculated as follows:

$$\begin{aligned} & {\text{Glycolysis}}\, = \,{\text{value before Oligo treatment}}-{\text{value before glucose treatment}}; \\ & {\text{Glycolysis capacity}}\, = \,{\text{value after Oligo treatment}}-{\text{value before glucose treatment}}; \\ & {\text{Glycolysis reserve}}\, = \,{\text{glycolysis capacity}}-{\text{glycolysis}}. \\ \end{aligned}$$

### Effect of mitochondrial dysfunction on L-MOF-agomir-mediated macrophage repolarization

RAW 264.7 cells were incubated with 100 ng ml^−1^ LPS for 24 h and then incubated on L-MOF-agomir titanium plates with 10 μM carbonyl cyanide 3-chlorophenylhydrazone (CCCP), 1 μM Oligo or 0.1 μM Rot for 72 h. The polarization states of the cells were examined by immunofluorescence staining and qRT-PCR.

### Establishment of rat peri-implantitis models

All animals were obtained from and kept in the Shanghai Jiao Tong University Affiliated Ninth People’s Hospital Animal Center (SYXK 2020-0025). Ethical approval for the study was provided by the Animal Care and Experiment Committee of Ninth People’s Hospital.

Sixty 10-week-old male Sprague–Dawley rats weighing approximately 350*g* were used in this study. Two rats were housed in a cage with sufficient water and food. The rats were divided into three groups on average: the L group, L-MOF group, and L-MOF-agomir group. The treatment was the same except for the implants in different groups. For peri-implantitis models, the anesthesia process, surgical procedures, and ligation-induced peri-implantitis were conducted as previously described. Briefly, the rats were anesthetized with sodium pentobarbital at a dose of 60 mg kg^−1^ intraperitoneally. The different groups of implants were placed in the rat immediately after extraction of the right maxillary first molar and the top of the implant was located 1 mm below the dentition plane. Four weeks later, a 3–0 silk thread was tied firmly around the implant neck with a loop for 2 weeks to induce peri-implantitis. After the surgery, 0.05 mg kg^−1^ buprenorphine was injected as an analgesic.

### In vivo macrophage polarization and bone regeneration evaluation around implant samples

After 2 weeks of ligation, the maxillary bone was collected and fixed with 4% paraformaldehyde immediately. After fixation, the samples were investigated using a micro-CT system (SkyScan 1176; Bruker micro-CT, Kontich, Belgium). The parameters for micro-CT imaging were as follows: source voltage, 65 kV; source current, 280 μA; AI 1.0-mm filter; pixel size, 18 μm; and rotation step, 0.55 degrees. The section through the center of the implant was selected from the sagittal and coronal planes as the measurement section. The vertical distance from the horizontal line of the implant top to the highest point of the osseo-implant contact surface was considered the measurement distance. Measurements were performed at the buccal, lingual, mesial and distal sites of the implant with DataViewer software (V.1.5.0.4 Bruker micro-CT, Kontich, Belgium). The range was 500 μm around the implant. CTAn (Bruker micro-CT, Kontich, Belgium) was used to complete the measurement and calculation of the bone mineral density (BMD), the percent bone volume (BV/TV), the trabecular thickness (Tb. Th) and the trabecular separation (Tb. Sp).

The lymphocyte aggregation, fiber and alveolar bone around the implant were observed with H&E staining and Masson staining. The maxillary bone was harvested and fixed with 4% paraformaldehyde immediately. After 48 h of fixation, the samples were rinsed with running water for 12 h. Then the implants were screwed out gently and the bone samples were decalcified in 20% EDTA. Samples were dehydrated in gradient concentrations of ethanol, permeabilized with xylene and embedded in paraffin. Serial sections across the samples were cut throughout the mesial-distal extension of the implant sites using a microtome (RM2235, Leica, Germany). Then the sections were stained with Masson staining and H&E staining.

The polarization states of macrophages in peri-implant sites were observed using immunofluorescence staining. Samples were processed to obtain serial sections as described above. Then, the sections were washed, permeabilized, and blocked incubated overnight at 4 °C with primary antibodies: rabbit monoclonal anti-iNOS antibody (ab178945, 1:200, Abcam) and mouse monoclonal anti-Arginase 1 antibody (1:200, sc-271430, Santa). Then the sections were incubated with Alexa Fluor 488-conjugated donkey anti-rabbit IgG (34206ES60, 1:200, Yeasen) and Alexa Fluor 594-conjugated donkey anti-mouse IgG (34112ES60, 1:200, Yeasen) secondary antibodies. The nuclei were stained with DAPI (40728ES03, 1:1000, Yeasen), and fluorescence images were obtained with a fluorescence microscope (Axio Scope A1, Zeiss). Six fluorescent sections were measured to determine the area ratio of positive cell staining according to the image gray threshold by ImageJ software (National Institutes of Health, NIH).

### Statistical analysis

The numerical data are presented as the mean plus/minus the standard deviation (mean ± SD). GraphPad Prism 8.0.3 statistical software was used for statistical analysis. The normal distribution of data was tested using the Shapiro–Wilk regular test. When normality was confirmed, one-way analysis of variance (ANOVA) was used for data with uniform variances to quantify differences, while for data with nonuniform variances, a nonparametric test was applied; *p < 0.05, **p < 0.01, ***p < 0.001.

## Results

### Characterization of MOF-agomir particles and L-MOF-agomir titanium materials

**Fig. 1:**
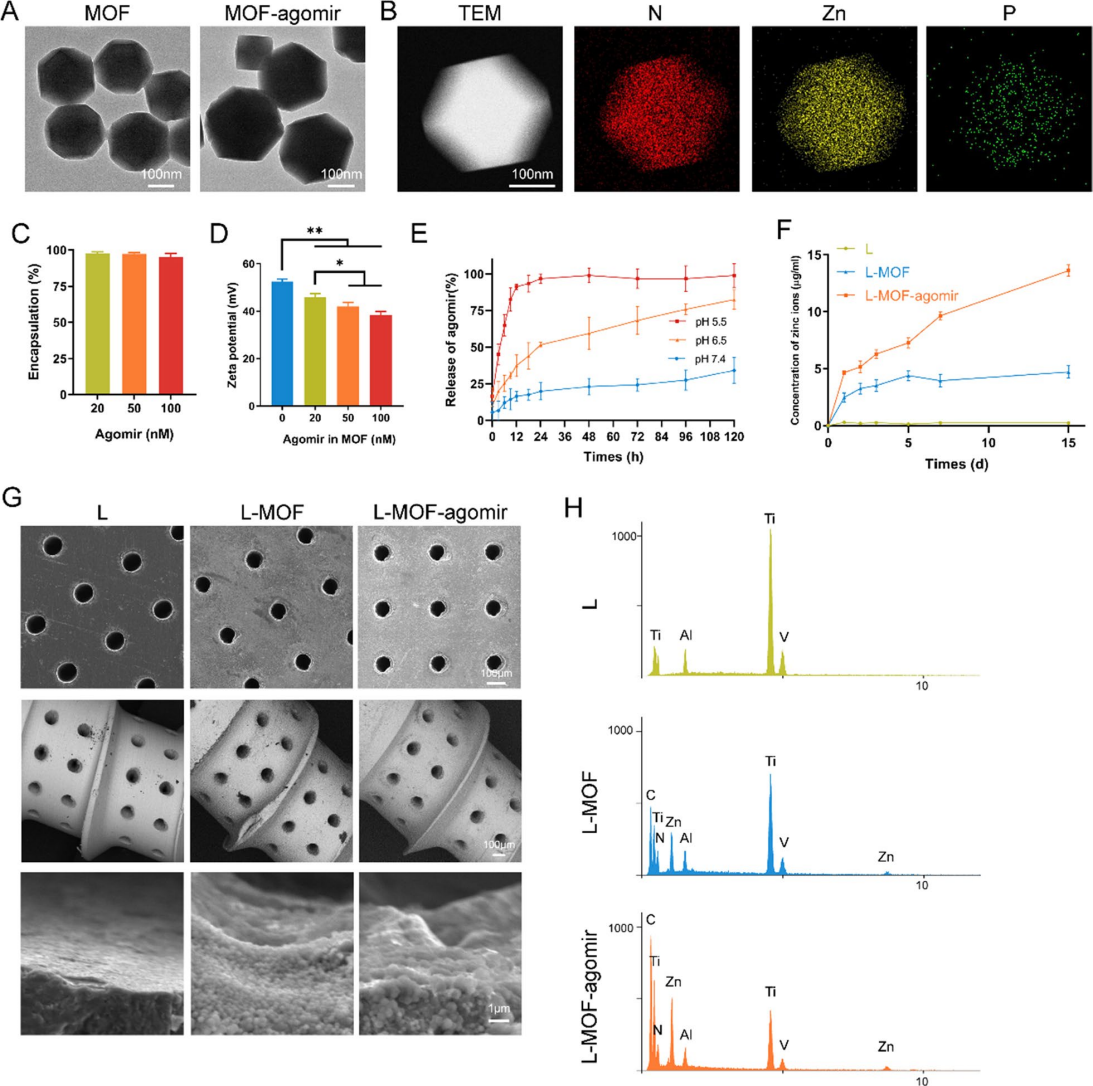
Characterization of MOF-agomir particles and L-MOF-agomir plates/implants. **A** Transmission electron microscopy images of MOF and MOF-agomir particles. **B** Energy dispersive spectroscopy images of N, Zn, P elements in MOF-agomir particles. **C** Encapsulation efficiency of MOF at different concentration of agomir (n = 3). **D** Zeta potential of MOF-agomir particles with various agomir inputs. (n = 3). **E** Agomir release from L-MOF-agomir plates at pH 5.5, pH 6.5 or pH 7.4 over time (n = 3). **F** Zinc ion release rate of L, L-MOF and L-MOF-agomir titanium plates in 10% FBS-containing DMEM. **G** Scanning electron microscope images of L, L-MOF and L-MOF-agomir implants. **H** Energy dispersive spectroscopy analysis of element composition in L, L-MOF and L-MOF-agomir implants

The composition and functional mechanism of the materials are shown in the Graphical Abstract. TEM images showed that the morphology of the MOF and MOF-agomir particles was regular, the edges of MOF particles were sharper and the density of MOF-agomir particles was higher (Fig. 1A). The size of MOF particles was approximately 200 nm, and that of MOF-agomir particles was approximately 240 nm (Additional file 1: Figure S1A). The EDS images of MOF-agomir particles showed the contents of N, Zn and P in MOF-agomir particles (Fig. 1B). There was no significant difference in the encapsulation efficiency in a certain concentration range. The encapsulation rate of 20 nM agomir in MOF was 97.8 ± 0.81%, that of 50 nM was 97.35 ± 1.02%, and that of 100 nM was 95.08 ± 2.45% (Fig. 1C, p > 0.05). The zeta potential of MOF in ddH_2_O was 52. 53 ± 1.03 mV, that of 20 nM agomir was 45.97 ± 1.53 mV, that of 50 nM was 42.00 ± 1.77 mV, and that of 100 nM was 38.33 ± 1.53 mV. The zeta potential test showed that the zeta potential of the MOF-agomir in ddH_2_O decreased with increasing agomir input concentration (Fig. 1D, p < 0.05). The fluorescence images showed that the agomir-Cy3 colocalized with MOF particles (Additional file 1: Figure S1B).

The release profile of the agomir from the L-MOF-agomir titanium plate was measured over time in PBS at different pH values. Sudden release of agomir was observed in pH 5.5 PBS group in 24 h, followed by sustained release. Where as in pH 7.4 PBS, sustained and slow release was observed. The released agomir was 19.82 ± 5.94% in supernatant of pH 7.4 PBS, 51.65 ± 1.67% in pH 6.5 and 96.74 ± 3.23% in pH 5.5 in 24 h. The release rate in 120 h was 34.16 ± 8.79% in pH 7.4, 82.47 ± 6.65% in pH 6.5 and 99.00 ± 8.22% in pH 5.5 (Fig. 1E). The release profile of the agomir from MOF-agomir nanoparticles was also measured (Additional file 1: Figure S1C). The results of zinc ion release from the L-MOF and L-MOF-agomir titanium plates showed that the zinc ions were slowly released into the culture medium without a burst-release effect. The highest concentration of zinc ions generated from the degraded L-MOF plates was 4.70 ± 0.56 μg/ml at 15 days. The L-MOF-agomir plates showed a higher concentration of 13.61 ± 0.50 μg/ml at 15 days (Fig. 1F). The morphology of the titanium materials was observed using SEM (Fig. 1G). Images showed homogeneous micropores with diameter of approximately 100 μm. More round and blunt edges, smaller pore size can be observed in the L-MOF and L-MOF-agomir groups. MOF particles uniformly cover the sidewall and edge of micropores, but the particle diameter was larger in the L-MOF-agomir group than that in the L-MOF group. Elemental carbon (C), nitrogen (N) and zinc (Zn) were found in the L-MOF and L-MOF-agomir groups, which demonstrated that the MOF particles and agomir were successfully incorporated into the titanium plates and implants (Fig. 1H).

### Incubation on L-MOF-agomir titanium induces macrophage M2 repolarization

**Fig. 2:**
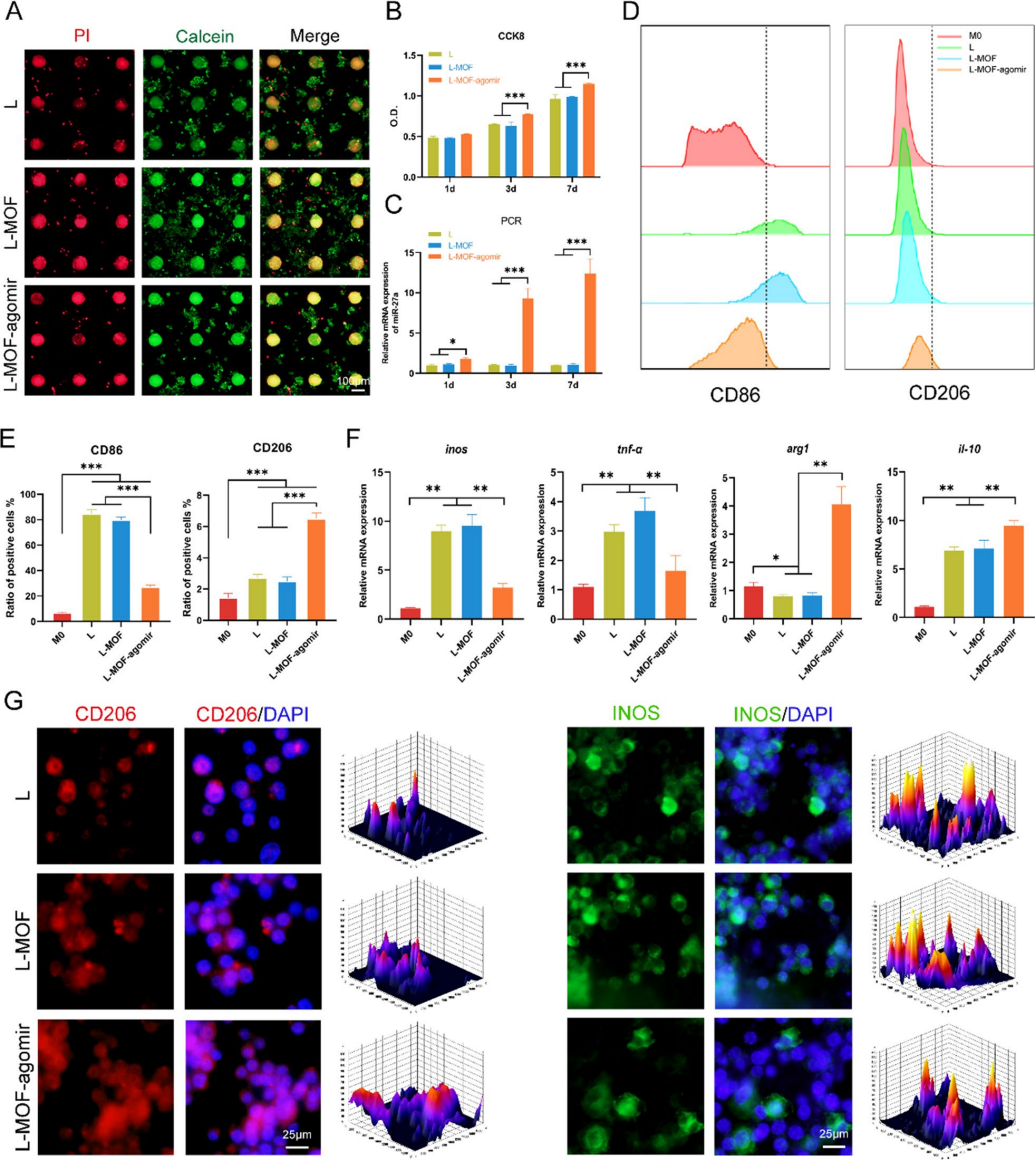
L-MOF-agomir titanium induced repolarization of macrophages from M1 into M2 phenotype after LPS stimulus in vitro. **A** Immunofluorescence images of macrophages incubated on different titanium plates (live/dead assay); live cells were stained with calcein-AM (green fluorescence), while dead cells were stained with PI (red fluorescence). **B** CCK8 assay was performed to measure the number of living cells. **C** Expression of miR-27a in macrophages incubated on different titanium plates. **D** Macrophages were stained with antibodies against the M1 surface marker CD86 and M2 surface marker CD206, followed by flow cytometric analysis. Representative histogram graphs are presented. **E** Statistical analysis of flow cytometry. **F** Expression of M1 polarization-related genes (*inos*, *tnf-α*) and M2 polarization-related genes (*arg1*, *il-10*) in macrophages incubated on different titanium plates (qRT-PCR). **G** Representative images of immunofluorescent staining showing M2 polarization-related marker CD206 and M1 polarization-related marker iNOS in macrophages. Data were shown as mean ± SD, * p < 0.05, ** p < 0.01, *** p < 0.001

The biocompatibility of titanium materials was examined by live/dead fluorescence staining (Fig. 2A, Additional file 1: Figure S2A). Green fluorescence represents living cells and red fluorescence represents dead cells. Images showed that in all three groups, living cells and dead cells were uniformly distributed, and the proportion of dead cells was low. SEM images suggested that BMSCs adhered to the surface of the titanium plates well in all groups, but there was a difference in the cell arrangement. Cells on L titanium were arranged in a shoal pattern, cells on L-MOF titanium were disorderly, and cells on L-MOF-agomir titanium were arranged in a vortex pattern (Additional file 1: Figure S2B). The CCK-8 assay results showed that the O.D. value in the L-MOF-agomir group was significantly higher than that in the L and L-MOF groups (Fig. 2B, Additional file 1: Figure S2C). PCR results showed that after 1, 3 and 7 days of culture on titanium plates, the expression of mir-27a in BMSCs and macrophages was significantly upregulated in the L-MOF-agomir group (Fig. 2C, Additional file 1: Figure S2D, p < 0.05). In addition, the antibacterial property of titanium was evaluated by live/dead staining. The L group had nearly complete live bacteria with green staining, while the L-MOF group and L-MOF-agomir group had substantial amounts of dead bacteria on top of each other (Additional file 1: Figure S3A). SEM images showed that the Porphyromonas gingivalis on the L-MOF and L-MOF-agomir titanium was shorter and fewer (Additional file 1: Figure S3B). Similarly, the absorbance in the L-MOF and L-MOF-agomir groups was lower than that in the L group (Additional file 1: Figure S3C). No significant difference was observed between the L-MOF and L-MOF-agomir groups.

To verify the immunomodulatory effects of L-MOF-agomir, the polarization states of macrophages incubated on titanium plates were assessed by immunofluorescence staining, flow cytometry and qRT-PCR. Flow cytometry showed that before LPS stimulation, macrophages expressed low levels of the M1 polarization marker CD86 and the M2 polarization marker CD206. After LPS stimulus, both M1 and M2 markers were increased. There were more CD86 positive macrophages in the L and L-MOF groups than in the L-MOF-agomir group. While, fewer CD206 positive macrophages were found in the L and L-MOF groups than those in the L-MOF-agomir group (Fig. 2D, E). Similarly, qRT-PCR results revealed that M2 polarization-related genes (*arg1* and *il-10*) were increased and M1 polarization-related genes (*inos* and *tnf-α*) were decreased in the L-MOF-agomir group (Fig. 2F). Immunofluorescence staining and fluorescence intensity distribution maps showed that after LPS stimulation and incubation on the L and L-MOF titanium plates, macrophages expressed relatively low levels of the M2 polarization marker CD206 and high levels of the M1 polarization marker iNOS. More macrophages highly expressed CD206, and fewer cells expressing the M1 polarization marker iNOS were observed in the L-MOF-agomir group (Fig. 2G).

### L-MOF-agomir titanium promotes osteogenesis by regulating macrophage repolarization

BMSCs were incubated on titanium plates directly and investigated with immunofluorescence staining (Additional file 1: Figure S4). There were many BMSCs growing around the edge of micropores in the L-MOF and L-MOF-agomir groups, and more osteogenic marker ALP was observed than that in the C and L groups.

**Fig. 3:**
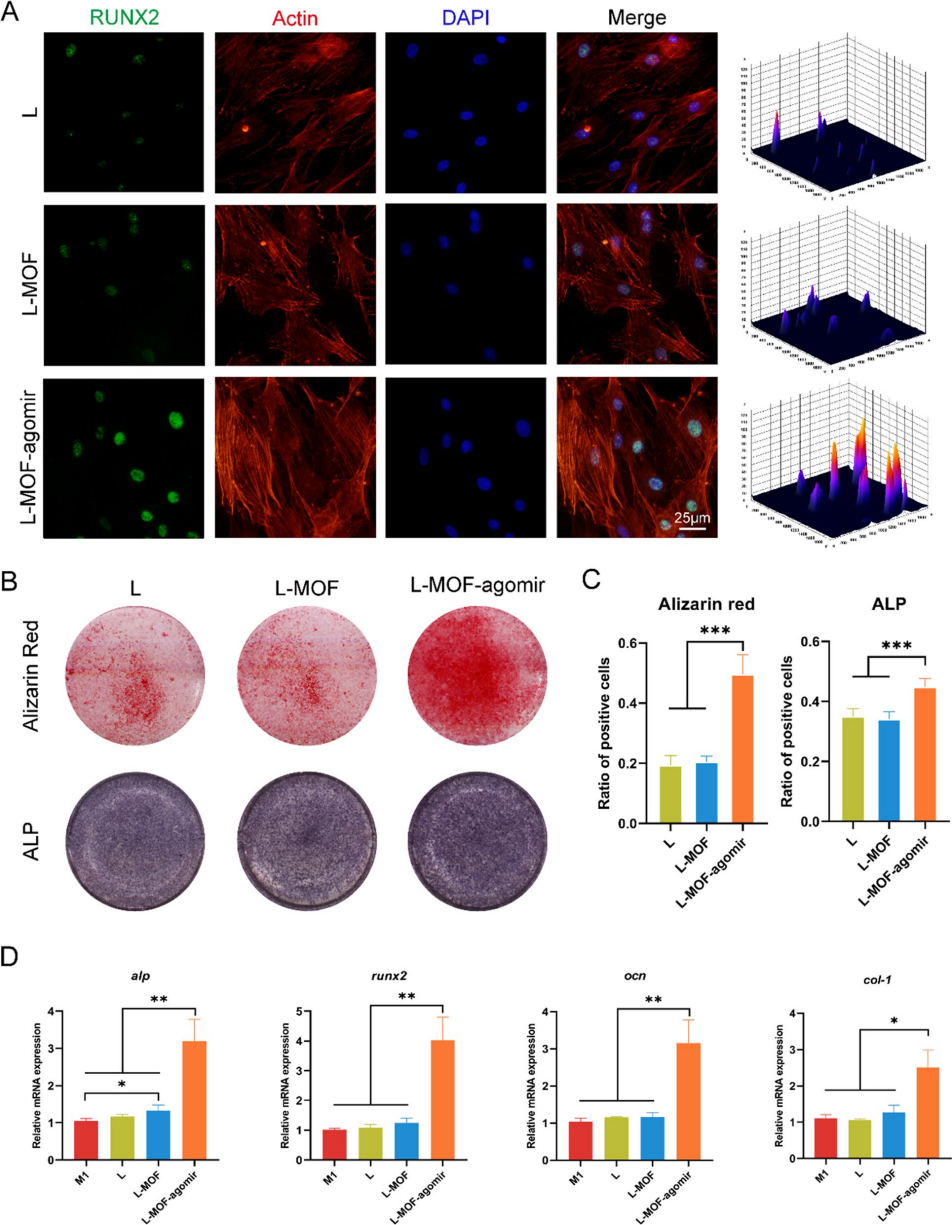
L-MOF-agomir titanium promotes immune osteogenesis by inducing macrophages M2 repolarization. **A** Representative images and distribution map of immunofluorescent staining showing osteogenesis marker RUNX2 in BMSCs cultured with macrophages supernatant in different groups. **B** Representative images showing the mineralization extracellular matrix and ALP staining of BMSCs in different groups. **C** The quantification of Alizarin Red and ALP staining. **D** Expression of osteogenesis-related genes (*alp, runx2, ocn, col-1*) in BMSCs (qRT-PCR). Data were shown as mean ± SD, * p < 0.05, ** p < 0.01, *** p < 0.001

The supernatant of macrophages incubated on different titanium plates was collected to culture BMSCs for immune osteogenic property investigation. Immunofluorescence staining and fluorescence intensity distribution maps showed that BMSCs expressed higher levels of the osteogenic marker RUNX2 in the L-MOF-agomir group than that in the L and L-MOF groups (Fig. 3A). Similarly, alizarin red staining and ALP staining showed that there were more mineralized nodules and ALP in the L-MOF-agomir group than those in the L and L-MOF groups (Fig. 3B, C). QRT-PCR results revealed that osteogenic-related genes (ALP, RUNX2, OCN, COL-1) were upregulated in BMSCs incubated with the L-MOF-agomir group macrophage supernatant, while there was no significant difference between the other groups (Fig. 3D).

### Incubation on L-MOF-agomir titanium improves mitochondrial morphology and promotes the metabolic transformation from glycolysis to OXPHOS in macrophage after LPS stimulus

**Fig. 4:**
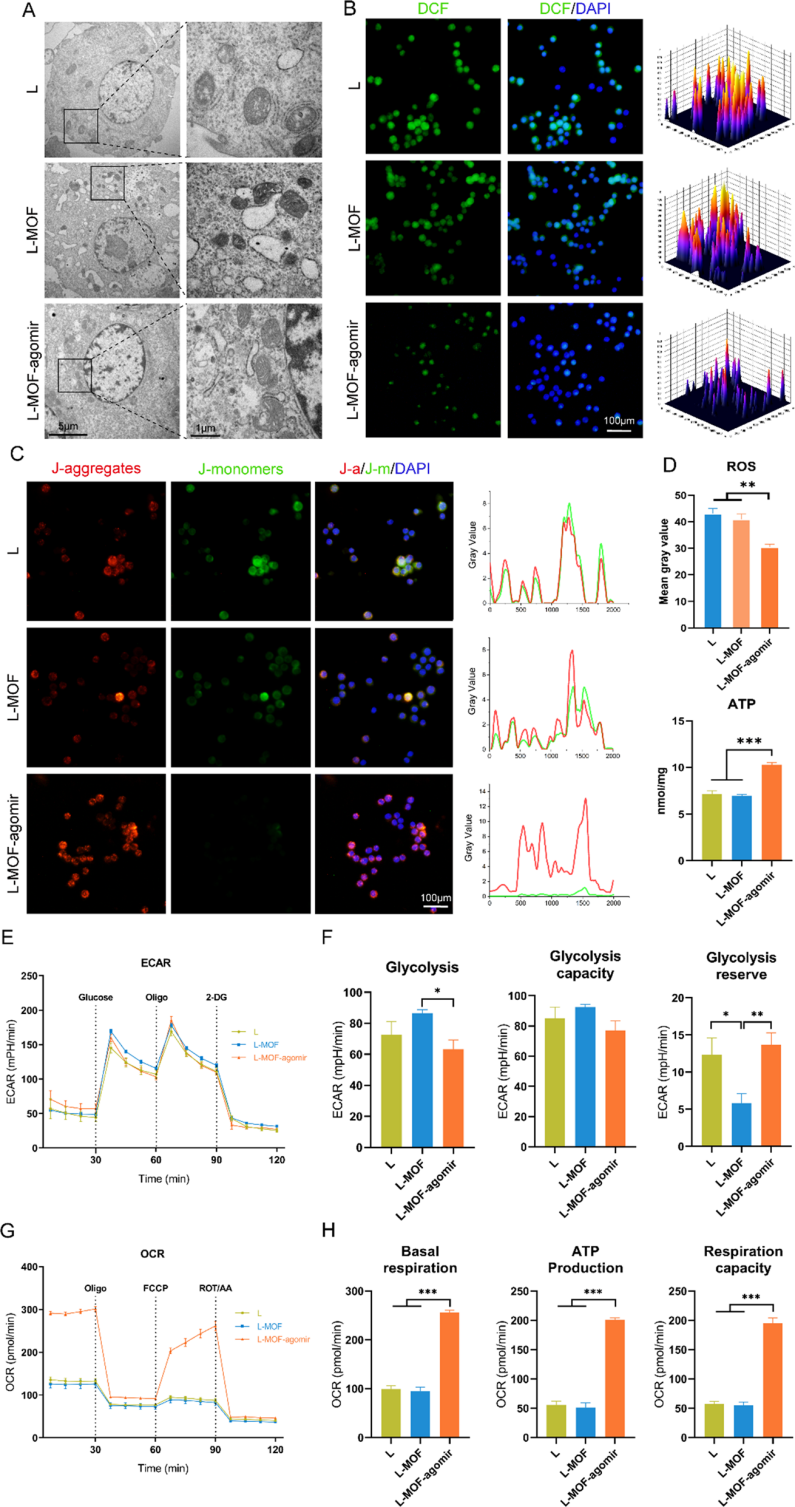
L-MOF-agomir titanium enhanced the mitochondrial function and promoted the metabolic transition from glycolysis to OXPHOS. **A** Representative TEM images showing the number and structure of mitochondria in macrophages after LPS stimulus incubated on different titanium plates. **B** Representative images and distribution map of immunofluorescent staining showing ROS in macrophages. **C** Representative images and fluorescence intensity histogram of immunofluorescent staining showing mitochondrial membrane potential in macrophages. **D** ROS and ATP production in macrophages. **E** Real-time ECARs of macrophages in different groups response to glucose, Oligo and 2-DG in 120 min. **F** Quantification of glycolysis, glycolytic capacity and glycolytic reserve. **G** Real-time OCRs of macrophages in different groups response to Oligo, FCCP and ROT/AA in 120 min. **H** Quantification of basal respiration, ATP production and respiration capacity. Data were shown as mean ± SD, * p < 0.05, ** p < 0.01, *** p < 0.001

Given that the O.D. value of CCK8 in macrophage incubated on L-MOF-agomir titanium was higher, while no significant difference was found in other cell activity experiments, we normalized the O.D. value to PI/Calcein fluorescence quantitation. Although the cck8 kit is often used to assess cell viability, it measures succinic dehydrogenase (SDH) activity, which is a rate-limiting enzyme of mitochondria. Given identical PI/Calcein fluorescence staining, the changes observed in the cck8 assay suggest metabolic differences in macrophage. The results showed that the O.D./Fluor value was higher in the L-MOF-agomir group than that in the L and L-MOF groups (Additional file 1: Figure S5). Hence, we further analyzed mitochondrial number and structure, ROS production, mitochondrial membrane potential (MMP) and ATP production. We found that there were fewer mitochondria in macrophages incubated on the L and L-MOF titanium compared to L-MOF-agomir after LPS stimulus. Mitochondrial swelling with the disappearance of mitochondrial cristae was frequently observed in the L and L-MOF groups, while the mitochondrial morphology was regular and the cristae were clearer in L-MOF-agomir group (Fig. 4A). The intracellular and mitochondrial ROS fluorescence staining showed that there was a lower DCF/DAPI ratio in the L-MOF-agomir group (Fig. 4B). Further quantitative analysis also showed that the ROS level was decreased in macrophages incubated on the L-MOF-agomir titanium (Fig. 4D). The JC-1 probe was used to detect the MMP in macrophages to investigate mitochondrial status. We found that there were obvious JC-1 monomers (green fluorescence) in the macrophages incubated on the L and L-MOF titanium, while more JC-1 aggregates were observed in the mitochondria of macrophages incubated on the L-MOF-agomir titanium. Gray value quantitative analysis suggested that the ratio of red/green fluorescence intensity was increased in the L-MOF-agomir group (Fig. 4C). Similarly, more ATP production was detected in the macrophages incubated on the L-MOF-agomir titanium (Fig. 4D).

As the polarization of macrophages is closely related to the respiratory metabolism states of cells, we used the seahorse test to detect the ECARs and OCRs of macrophages incubated on titanium. Although the ECARs curve was similar among the different groups (Fig. 4E), the cells incubated on the L-MOF-agomir titanium had significantly lower glycolysis than the cells on other titanium (Fig. 4F, p < 0.05). The glycolysis reserve was lower in cells on L-MOF titanium than on other titanium. The trend of glycolysis capacity seemed to be similar to that of glycolysis, but there was no significant difference among the groups. In parallel, the OCR curve was drawn based on the cell mitochondrial stress test to calculate the OXPHOS parameters of macrophages. The OCR curve of macrophages incubated on the L-MOF-agomir titanium was significantly different from those incubated on other titanium (Fig. 4G). The basal respiration, ATP production and respiration capacity in macrophages incubated on the L-MOF-agomir titanium were significantly higher than those in cells on other titanium (Fig. 4H, p < 0.001). There was no significant difference between cells on the L-MOF titanium and cells on the L titanium (p > 0.05).

**Fig. 5:**
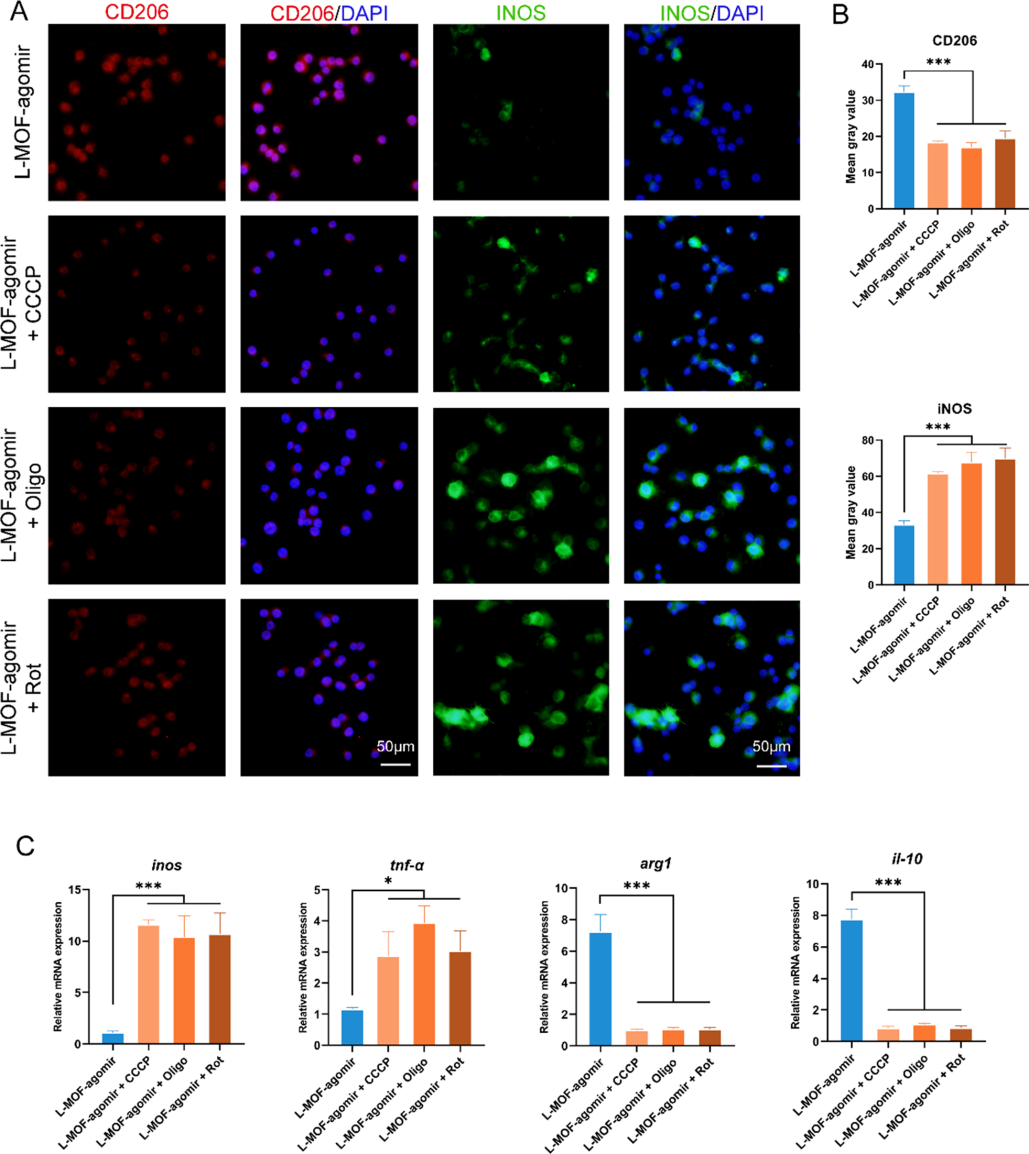
Incubated on the L-MOF-agomir titanium plates with OXPHOS inhibitor abrogated the macrophage repolarization function. **A** Representative images of immunofluorescent staining showing M2 polarization-related marker CD206 and M1 polarization-related marker iNOS in macrophages. **B** Mean gray value of immunofluorescent intensity. **C** Expression of M1 polarization-related genes (*inos*, *tnf-α*) and M2 polarization-related genes (*arg1*, *il-10*) in macrophages (qRT-PCR)

To verify the key role of metabolic transformation in L-MOF-agomir-induced macrophage repolarization, we incubated macrophages on L-MOF-agomir titanium plates with the OXPHOS inhibitors CCCP, Oligo and Rot. Immunofluorescence staining showed less green fluorescence (iNOS) and more red fluorescence (CD206) in the L-MOF-agomir group (Fig. 5A). Further quantitative analysis showed that the mean gray value of CD206 in L-MOF-agomir with either CCCP, Oligo or Rot significantly decreased while the mean gray value of iNOS increased (Fig. 5B, p < 0.001). In addition, qRT-PCR results indicated that the addition of inhibitors significantly increased the expression of *inos* and *tnf-α* and decreased the expression of *arg1* and *il-10* in macrophages incubated on the L-MOF-agomir titanium plates (Fig. 5C, p < 0.05).

### L-MOF-agomir implants induce macrophage to M2 polarization and relieve peri-implantitis bone resorption in vivo

**Fig. 6:**
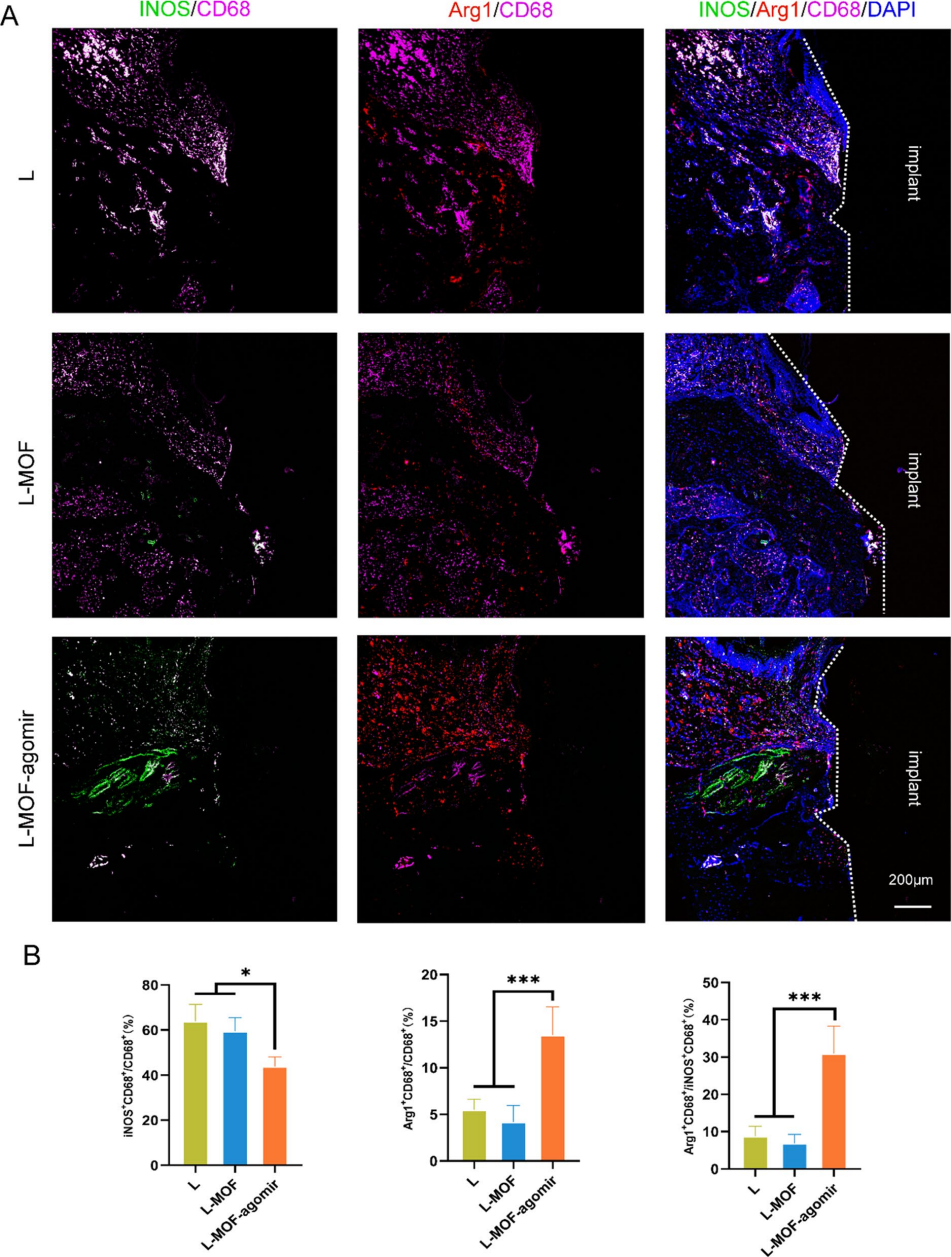
L-MOF-agomir implants promotes recruited macrophages toward M2 polarization in vivo. **A** Representative immunofluorescent staining of M1 polarization-related markers (iNOS/CD68) and M2 polarization-related markers (Arg1/CD68) in macrophages around the implant after 2-week ligation. **B** Quantification and statistical analysis of immunofluorescent positive area. Data were shown as mean ± SD, * p < 0.05, ** p < 0.01, *** p < 0.001

After the implantation surgery, a total of 10 implants were lost, 4 in the L group, 3 each in the L-MOF group and L-MOF-agomir group. All the remaining implants achieved good soft tissue closure and were stable 4 weeks after surgery. No implant loss was found after ligation. The immune status around the implant was evaluated with immunofluorescence staining. The fluorescence gray value distribution map showed the intensity of red fluorescence and green fluorescence at different locations (Fig. 6A). Specific molecules (iNOS for M1, Arg1 for M2) were used to identify the polarization states of macrophages around the implants 2 weeks after ligation. Immunofluorescence staining and quantitative analysis revealed a higher ratio of Arg1/DAPI around the L-MOF-agomir implants than that around other implants, while a lower ratio of iNOS/DAPI was observed around the L-MOF-agomir implants than that around the L-MOF implants. There was no significant difference in the ratio of iNOS/DAPI around the L implants and that around other implants. Correspondingly, the ratio of iNOS/Arg, which represents the ratio of M1/M2 polarization, was lowest around the L-MOF-agomir implants (Fig. 6B, p < 0.05).

**Fig. 7:**
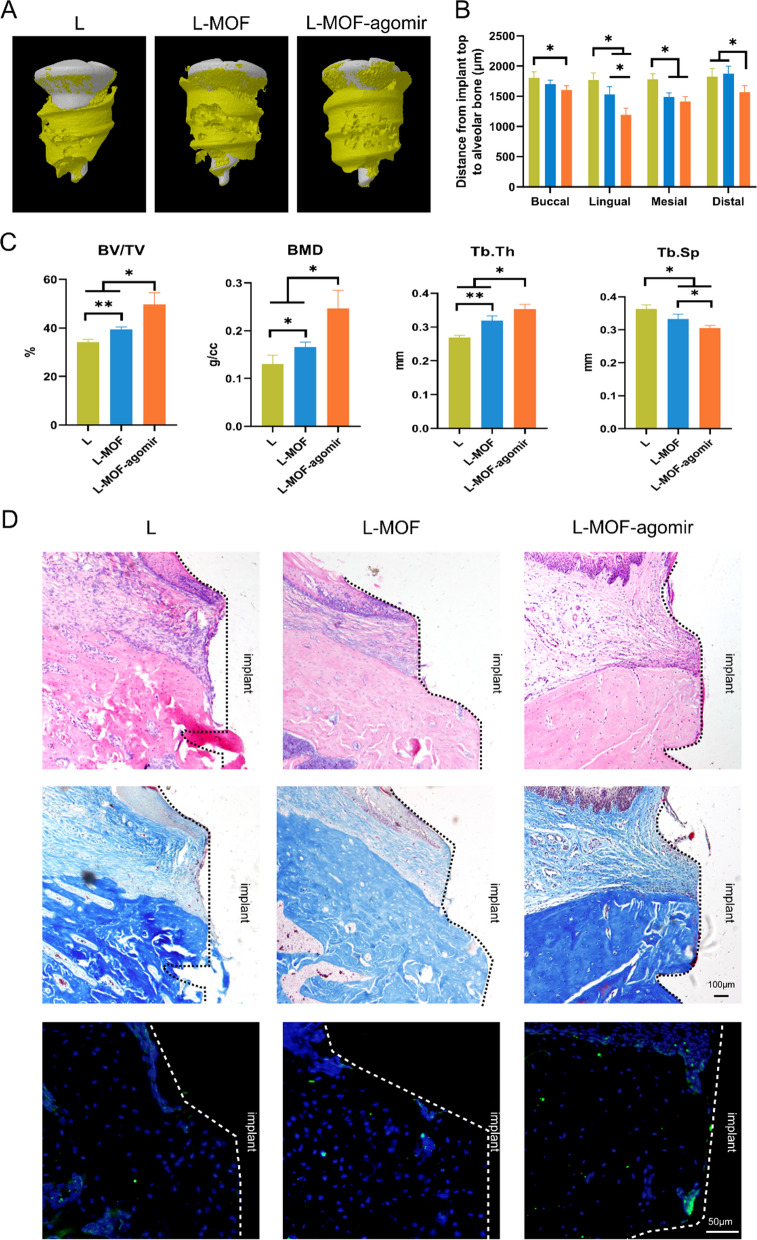
L-MOF-agomir implants inhibited ligation induced bone resorption around the implant. **A** Representative of 3D reconstruction of the bone around the implant after two weeks of ligation. **B** Distance from the implant top to the highest point of alveolar bone around the implant. **C** Quantitative analysis of BV/TV, BMD, Tb.Th and Tb.Sp at 2 weeks after ligation based on micro-CT scanning. **D** Representative images of H&E, Masson, and anti-RUNX2 immunofluorescence staining of tissue around implants. The implant is indicated by a dotted line. Data were shown as mean ± SD, * p < 0.05, ** p < 0.01

Next, we investigated bone resorption around the implant. Micro-CT scanning showed that new bone was formed around the implants. The bone around the neck of the L implants and the L-MOF implants showed wedge-shaped resorption. The bone around the L-MOF-agomir implants was denser and more complete, and there was no obvious resorption of the bone around the implant neck (Fig. 7A). The distance from the implant top to the highest alveolar bone was investigated to reflect the degree of bone resorption. The bone resorption degree was lower around the L-MOF-agomir implants than that around other implants on the lingual and distal sites. On the buccal and mesial sites, the bone resorption around the L-MOF-agomir implants was significantly lower than that around the L implants, but there was no significant difference between L-MOF-agomir and L-MOF implants (Fig. 7B). For histomorphometry of alveolar bone around the implant, L-MOF-agomir implants had significantly highest BV/TV, BMD, Tb. Th and lowest Tb. Sp among the 3 tested groups (L, L-MOF and L-MOF-agomir) at 2 weeks after ligation. The BV/TV, BMD, Tb. Th were also higher and the Tb. Sp was lower in the L-MOF implant group than those in the L implant group (Fig. 7C, p < 0.05). Tissue decalcification sections with H&E staining showed that there were more inflammatory cells and wedge-shaped bone resorption around the L implants. Bone tissue was porous with many inflammatory cells infiltrating the vessels. Fewer inflammatory cells infiltrating the vessels and mild bone resorption around the implant were observed in the L-MOF implant group. In the L-MOF-agomir implant group, the soft tissue showed good closure with the implant, with a small number of inflammatory cells confined to the ligation sites. The bone tissue margin was relatively complete with little bone absorption, vascular hyperplasia, and inflammatory cell infiltration. Masson staining was used to display the collagen fiber around the implants, which was dyed blue. The collagen fiber was orderly and complete around the L-MOF and L-MOF-agomir implants, while the fiber was broken and disordered around the L implants. Furthermore, more RUNX2 (marked by AF488) could be observed around the L-MOF-agomir implants (Fig. 7D).

## Discussion

Although peri-implantitis and periodontitis have many similarities in some aspects, many clinical treatments that are effective for periodontitis are proving to be less effective for peri-implantitis [28, 29]. With the development of novel biomaterials and advanced processing technologies, many oral implants have been developed to promote bone regeneration and inhibit bone resorption around implants [30, 31]. Most previous studies have emphasized the role of implant biomaterials in mesenchymal stem cells, osteoblasts, and bacteria, but ignored the important role of immune balance [32–35]. Over the past few decades, studies have found that macrophages are not only essential in the immune system, but also involved in the process of bone remodeling, and the dynamic transformation of macrophage M1/M2 polarization at the right time plays a key role in the progression of osteoclast/osteogenesis [36, 37]. As an inflammatory bone resorption disease, peri-implantitis is characterized by progressive destruction of the soft and hard tissues surrounding the implant, in which macrophages have been reported to aggregate in large numbers in the peri-implantitis tissues and interact with mesenchymal stem cells [38]. Therefore, implant surface modification from “immune-inert” to “immunomodulatory” is an attractive idea, which may regulate the immune balance around the implant, prevent or alleviate bone resorption, and promote bone regeneration in peri-implantitis.

Here, we designed a micro-nano composite coating implant with a pH-responsive controlled drug release function. The micropores were performed by femtosecond laser, which has been widely used to prepare microscopic patterns on the surface of titanium [39]. The femtosecond laser can directly generate micro-nano scale structures on the surface of material due to its extremely short pulse width. Studies have found that femtosecond laser modification can change the hardness, roughness, and wettability of metals, which also affect titanium surface biological activity and early osseointegration [40]. In addition, micropores with 100 μm have been reported to have good bone conductivity. This may be because it mimics the porous structure of natural bone, which has osteons and vascular channels 100 μm in diameter [41]. The nanostructure and pH-responsive controlled drug release function were achieved by MOF coating. Although studies have focused on the preparation of MOF nanoparticles, the preparation of MOF-based membranes of films on metal surface has also been reported. Hermes et al. [42] fabricated MOF-5 film on gold surface; Wang et al. [43] prepared ZIF-8 film on porous titanium with self-assembled monolayers. In our work, the self-assembled MOF coating we synthesized was ZIF-8, which has been found to have osteogenic and antibacterial properties [43]. The inner and bottom walls of the holes on titanium drilled by the femtosecond laser were rough, which provided the binding site of self-assembled monolayer structures. Moreover, the design of coating in the hole avoids the problem of coating stripping during the implantation. To encapsulate miR-27a-agomir within the nanoscale MOFs, an in situ biomineralization method was used [44]. The EDS images of MOF-agomir and fluorescence colocation images of MOF-agomir-Cy3 suggested the loading of agomir in MOF. The diameter of MOF-agomir particles was larger than that of MOF particles, and the zeta potential was decreased after agomir loading, which may be due to the premix of agomir increasing the complexity of the system and increasing the difficulty of charge balance. With increasing agomir load, the negatively charged agomir decreased the surface zeta potential of the positively charged MOF, which also confirmed the loading of the agomir [45]. The encapsulation efficiency results showed that miRNA-agomir could be incorporated with high efficiency over a wide range of inputs. The strong loading capacity of MOFs was likely attributed to physical confinement of the nanoporous structure, as well as electrostatic interactions between the framework’s metal ion and the miRNA’s phosphates [46]. In addition, the special structure of MOFs gives them special pH responsiveness, which could remain intact until acute inflammation occurs, in which the mildly acidic environment would trigger agomir release. The MOF nanoparticles could also be internalized by cells. Wan et al. found that internalized nano ZIF-8 could release Zn^2+^ inside rBMSCs and the intracellular ions would also synergistically alter osteogenesis. In our work, the internalized MOF-agomir could also release agomir-27a, which may play a key role in macrophage repolarization.

To investigate the effect of different titanium materials on cells, we cultured macrophages and BMSCs directly on titanium plates. Interestingly, the BMSCs on the L-MOF-agomir titanium plates were arranged in swirl, like Haversian system. Furthermore, more osteogenic marker-ALP was observed in the BMSCs on the L-MOF-agomir titanium, which may be due to the osteogenic effect of miR-27a and Zn^2+^ in MOF particles [47, 48]. The osteogenic effect of Zn^2+^ was consistent with many studies, which suggested that Zn^2+^ effectively promotes the expression of ALP and RUNX2 to promote osteogenesis. Our previous study found that miR-27a could effectively improve osteogenesis under TNF-α condition, and in the present study we combined the two to achieve better bone-promoting effect. In addition, the antibacterial ability of L-MOF and L-MOF-agomir titanium may also be due to the Zn^2+^ in MOFs. As has been reported, Zn^2+^ can be transported across bacterial membranes via ion channels, expending energy and inducing a disorder effect on bacteria [49]. Most importantly, the L-MOF-agomir titanium exhibited powerful immunomodulatory effects, which could inhibit M1 polarization and promote M1 to M2 repolarization in macrophages after LPS stimulation. Given the large number of M1 macrophages in the inflammatory area, whether the immunomodulatory effect of promoting M0 to M2 polarization is effective has been debated, and the repolarization of M1 to M2 is difficult. M1 macrophages fail to convert into M2 cells upon IL-4 exposure in vitro and in vivo. In contrast, M2 macrophages are more plastic and readily repolarized into the M1 state. Although many biomaterial strategies effectively modulate the direction of macrophage polarization, the repolarization of M1 to M2 appears to be the current challenge. In our studies, LPS-induced M1 polarization was used to simulate an inflammatory environment in vitro, and the repolarization from M1 to M2 was investigated. The decreased expression of M1 polarization markers (iNOS, CD86, TNF-α) and the increased expression of M2 polarization markers (Arg1, CD206, IL-10) in macrophages incubated on the L-MOF-agomir titanium confirmed successful repolarization. No significant difference was observed in the L-MOF group, suggesting that miR-27a may be the key to immunomodulatory functions. Some previous studies have found that the expression of miR-27a in LPS-induced RAW264.7 macrophages was downregulation, and overexpression of miR-27a alleviated LPS-induced acute lung injury in mice via inhibiting inflammation and apoptosis through modulating TLR4/MyD88/NF-κB pathway [19]. However, the role and mechanism of miR-27a in the polarization of macrophages are not completely clear. Recent advances highlight a central role of the immunometabolism profile in the activation status and associated functions of macrophages [50], and factors affecting macrophage metabolism can be used to modulate macrophage polarization [51]. In our studies, we found that the O.D./Fluor value of the CCK8 test in the L-MOF-agomir group was highest, which suggested higher SDH activity, a rate-limiting enzyme of mitochondria, in the L-MOF-agomir group. Therefore, we focused further research on mitochondria, and found that the L-MOF-agomir titanium can attenuate the structure and number of mitochondrial disorders caused by LPS. The recovery of mitochondrial functions further promotes the TCA cycle. The recovery of succinate dehydrogenase activity reduced the accumulation of succinic acid, which is a widely known proinflammatory metabolite that accumulates in LPS-induced M1 macrophages [52]. Succinate dehydrogenase catalyzes the conversion of succinic acid to fumaric acid, which has broad anti-inflammatory and antioxidant activities, inducing a decreased in ROS production [53]. In addition, the OXPHOS inhibitors CCCP (an OXPHOS uncoupler), Oligo (an ATP synthesis inhibitor) and Rot (an electron transport chain inhibitor) can abrogate the macrophage repolarization effects of L-MOF-agomir. In general, our data suggest that the L-MOF-agomir titanium can alleviate mitochondrial damage and promote the transition from glycolysis to OXPHOS, which may promote the macrophage repolarization of M1 to M2.

Macrophages are major regulators of all stages of bone remodeling, and the crosstalk between macrophages and BMSCs plays an important role [54]. Although the role of macrophage polarization in the process of bone remodeling is still debated, the importance of the balance and transformation of macrophage polarization in proper time is unquestionable. Inflammatory M1-like macrophages present during the early stages of bone remodeling contribute to bone formation. Inflammation-regulated M2-like macrophages can contribute to the bone formation by MSCs during the late stages of the bone remodeling. However, in the inflammatory environment of peri-implantitis, the excessive infiltration of M1 macrophages and inflammatory factors inhibits the osteogenic differentiation of BMSCs and induces tissue damage [55]. Therefore, the strategy of regulating hyperactive M1-macrophage polarization and promoting M1-M2 repolarization to initiate the next stage of bone remodeling has attracted much attention in the inflammatory bone defect field. In our studies, we found that macrophages incubated on L-MOF-agomir titanium plates had a higher ability to promote BMSCs osteogenesis. Although L-MOF-agomir titanium has a direct osteogenic effect, we detached macrophages from titanium plates and recultured them in new complete medium to avoid the influence of residual components in the supernatant. The immune osteogenesis of L-MOF-agomir titanium may be due to the change of the M1/M2 polarization ratio, as well as the cytokines in the medium, thus affecting the behavior of stem cells, which was consistent with some previous studies [56].

To explore the effect of different implants on peri-implantitis in vivo, we established a rat model of ligation-induced peri-implantitis. A well-established animal model can increase the convenience of the experiment, improve credibility, and promote research on the pathogenesis and treatment of human diseases [57]. Although dogs are commonly used as animal models in the field of dental implants, much recent research has turned to rodents [58, 59]. Compared with large animals, small animals have shorter modeling times, lower rearing costs, small differences between individuals, and more suitable experimental reagents. Previous studies have demonstrated the feasibility of dental implants and peri-implantitis in rat models [60]. However, the rat model still has some unavoidable shortcomings, such as the difficulty of surgery and the need for customized implant systems due to its small size. In the present study, we successfully established a rat peri-implantitis model and found that the L-MOF-agomir implants could alleviate bone resorption around the implants during the ligation period, which may be due to both the regulation of macrophage polarization and the bone-promoting effect. In addition, antibacterial function was also beneficial for the relief of peri-implantitis.

## Conclusions

In summary, our study prepared L-MOF-agomir implants for the first time, and we found that the implant could effectively promote macrophage M1 to M2 repolarization, promote bone regeneration, and alleviate ligation-induced peri-implantitis inflammation and bone resorption in rats. We further found that the mechanism of macrophage repolarization may target the structure and function of mitochondria and promote the metabolic transition from glycolysis to OXPHOS. Our work represents an important step for the design of biomultifunctional implants and the treatment of peri-implantitis, and provides a new idea for the regulation of peri-implantitis immune metabolism and immune osteogenesis. Further exploration of the role of metabolic regulation in various cells and more detailed molecular mechanisms may provide a new perspective for the development of novel multifunctional implants.

### Supplementary Information

**Additional file 1:** Supplementary figures.

**Additional file 2:** Supplementary tables.

## Data Availability

The data that support the findings of this study are available from the corresponding author upon reasonable request.
