# Supplementary material for: Reprogramming mitochondrial metabolism of macrophages by miRNA-released microporous coatings to prevent peri-implantitis
Source: J Nanobiotechnology. 2023 Dec 17;21:485. doi: 10.1186/s12951-023-02244-z (PMC10726513; doi:10.1186/s12951-023-02244-z)
Supplement: Supplementary file 1 — Additional file 1: Supplementary figures. [file 12951_2023_2244_MOESM1_ESM.docx]

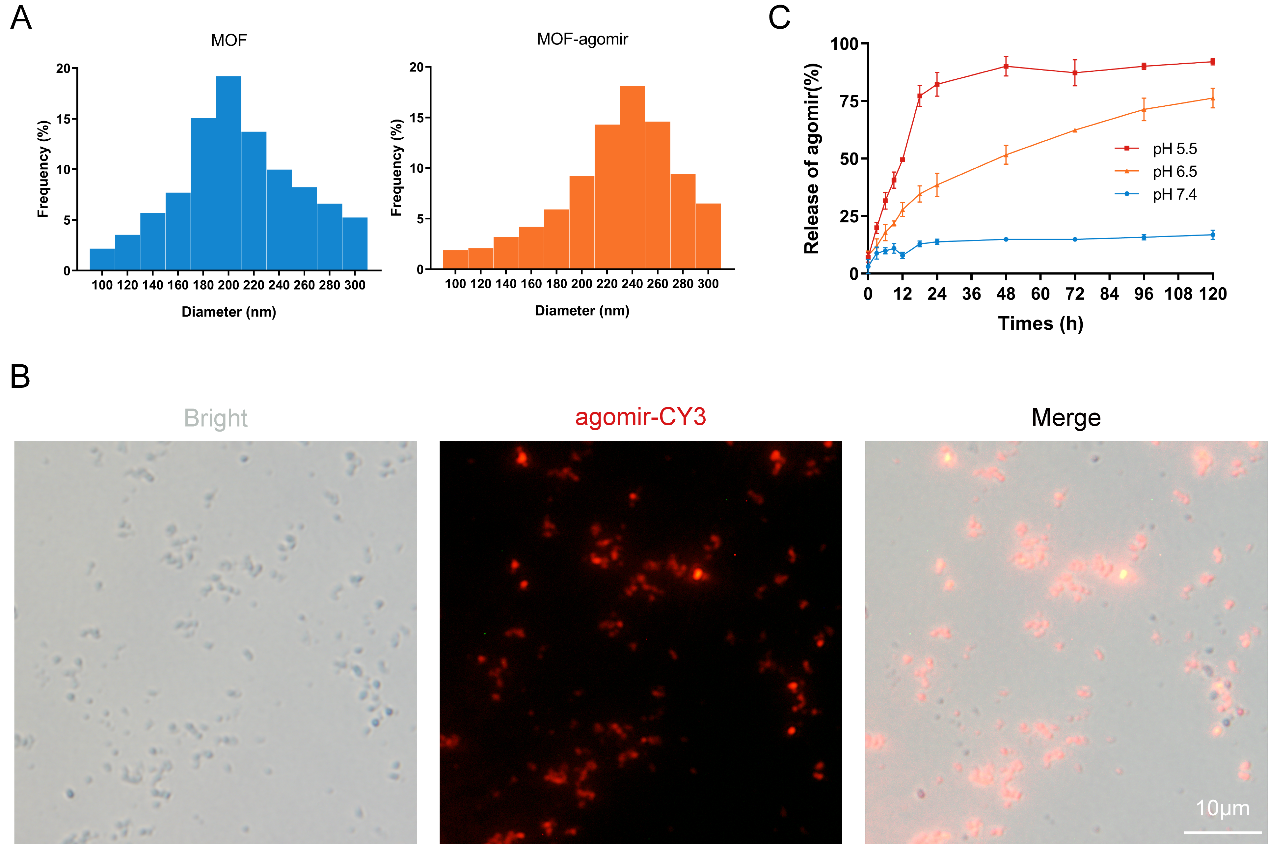


Figure S1 Supplemental characterization of MOF-agomir particles. (A) Diameter frequency of MOF and MOF-agomir particles. (B) MOF-agomir fluorescence co-localization. (C) Agomir release from MOF-agomir at pH 5.5, pH 6.5 or pH 7.4 over time (n=3).


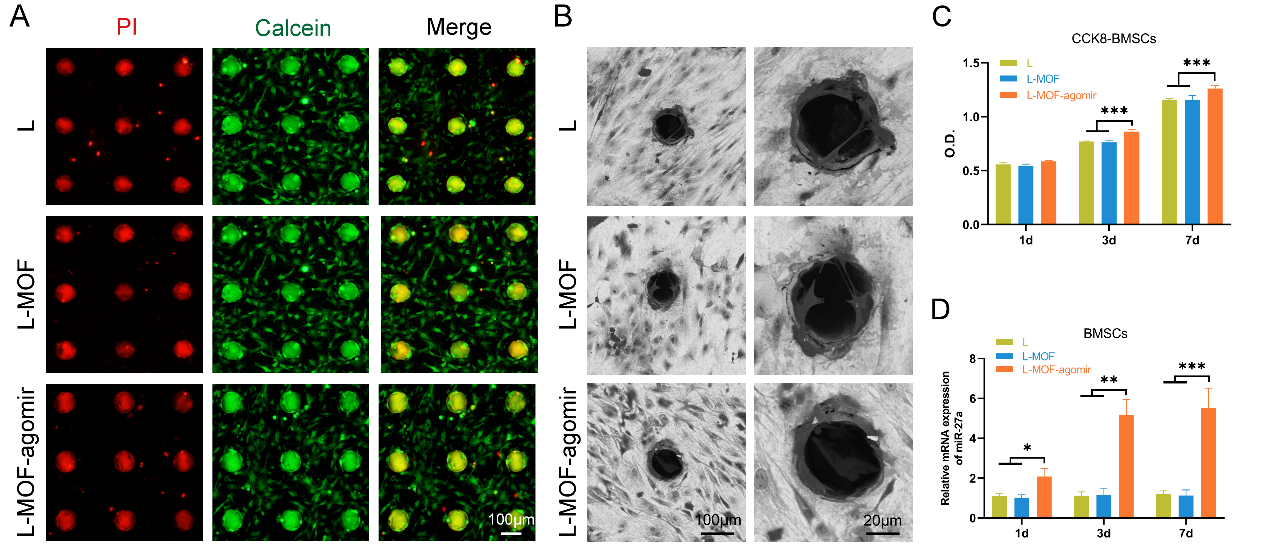


Figure S2 BMSCs incubated on the L-MOF and L-MOF-agomir titanium plates. (A) Immunofluorescence images of BMSCs incubated on different titanium plates (live/dead assay); live cells were stained with calcein-AM (green fluorescence), while dead cells were stained with PI (red fluorescence). (B) SEM images of BMSCs incubated on different titanium plates. (C) CCK8 assay was performed to measure the number of living cells. (D) Expression of miR-27a in BMSCs incubated on different titanium plates. Data were shown as mean ± SD, * p < 0.05, ** p < 0.01, *** p < 0.001.


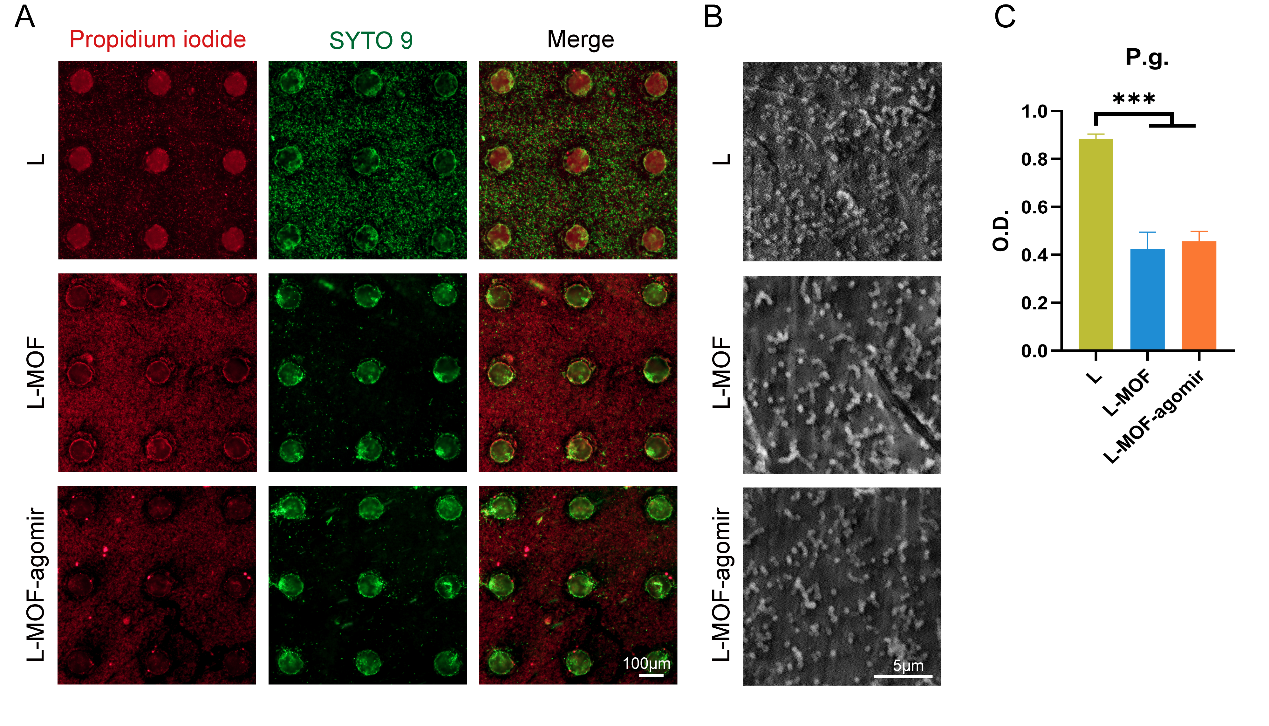


Figure S3 The L-MOF and L-MOF-agomir titanium plates inhibited the P. g. growth. (A) Representative immunofluorescence images of P. g. incubated on different titanium plates (live/dead assay); live bacteria were stained with SYTO9 (green fluorescence), while dead bacteria were stained with Propidium iodide (red fluorescence). (B) SEM images of P. g. incubated on the L, L-MOF and L-MOF-agomir titanium plates. (C) The number of bacteria was measured by ultraviolet spectrophotometer. Data were shown as mean ± SD, *** p < 0.001.


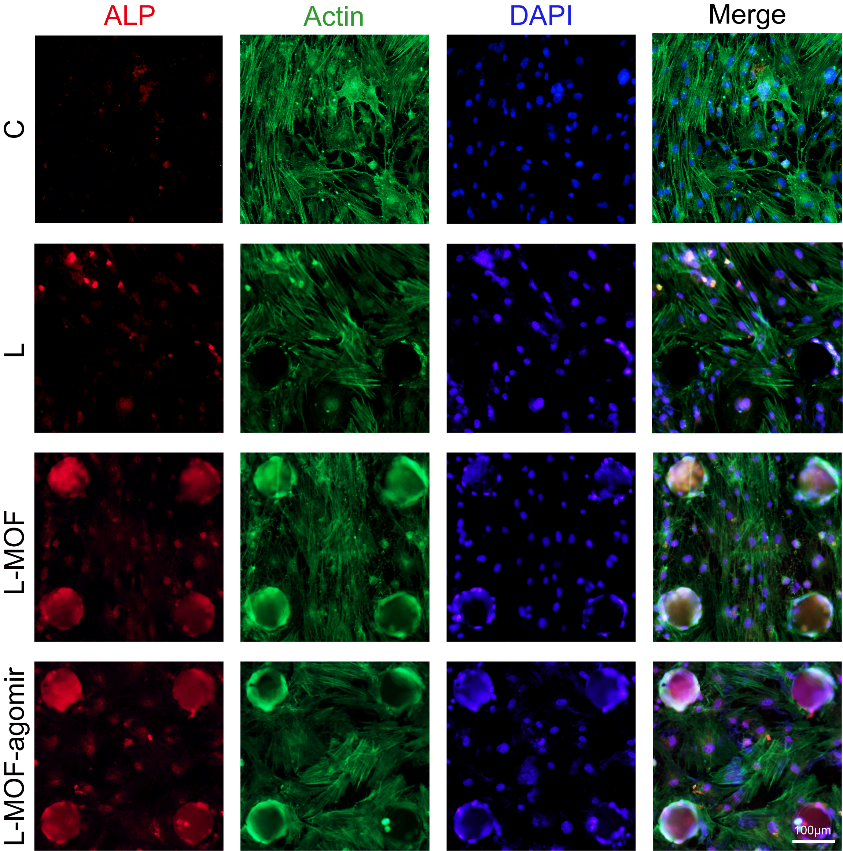


Figure S4 Osteogenic effect of titanium plates with different treatments. Representative images of immunofluorescent staining showing osteogenesis-related marker ALP (shown in red), and cytoskeleton (shown in green) in BMSCs.


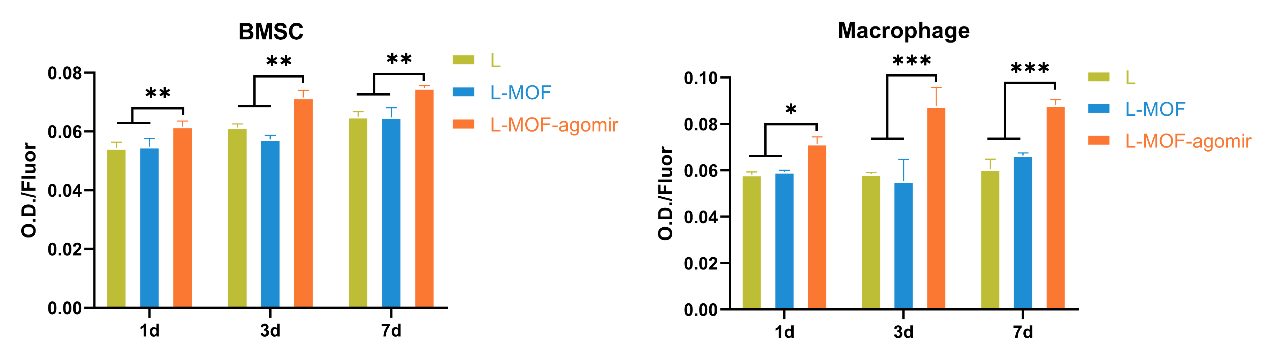


Figure S5 CCK8 was identified by fluorescence area of living cells in BMSCs and macrophages. Data were shown as mean ± SD, * p < 0.05, ** p < 0.01, *** p < 0.001.
