# Supplementary material for: Reprogramming mitochondrial metabolism of macrophages by miRNA-released microporous coatings to prevent peri-implantitis
Source: J Nanobiotechnology. 2023 Dec 17;21:485. doi: 10.1186/s12951-023-02244-z (PMC10726513; doi:10.1186/s12951-023-02244-z)
Supplement: Supplementary file 2 — Additional file 2: Supplementary tables. [file 12951_2023_2244_MOESM2_ESM.docx]

Table S1 Primers used for qRT-PCR

| Gene | Forward primer sequence | Reversed primer sequences |
| --- | --- | --- |
| *miR-27a* | GGGTTCACAGTGGCTAA | CAGTGCGTGTCGTGGAGT |
| *u6* | CAATACAGAGGAGATTAGCATGG | GTTTCACAAATTTGCGTGTCA |
| *inos* | ACTCAGCCAAGCCCTCACCTAC | TCCAATCTCTGCCTATCCGTCTCG |
| *tnf-α* | GCCTCTTCTCATTCCTGCTTGTGG | GTGGTTTGTGAGTGTGAGGGTCTG |
| *arg1* | CTCCAAGCCAAAGTCCTTAGAG | AGGAGCTGTCATTAGGGACA |
| *il-10* | GCTCTTACTGACTGGCATGAG | CGCAGCTCTAGGAGCATGTG |
| *alp* | GCCTACTTGTGTGGCGTGAA | AGGATGGACGTGACCTCGTT |
| *runx2* | TCCGCCACCACTCACTACCAC | GGAACTGATAGGACGCTGACGAAG |
| *ocn* | GGACCCTCTCTCTGCTCACTCTG | ACCTTACTGCCCTCCTGCTTGG |
| *col-1* | CGAGTCACACCGGAACTTGG | CCAATGTCCAAGGGAGCCAC |
| *β-actin* | CATCCGTAAAGACCTCTATGCCAAC | ATGGAGCCACCGATCCACA |
